# Extracellular Vesicles as Epigenetic Regulators of Redox Homeostasis: A Systematic Review and Meta-Analysis

**DOI:** 10.3390/antiox14050532

**Published:** 2025-04-29

**Authors:** Cristina Mas-Bargues, Javier Huete-Acevedo, Marta Arnal-Forné, Laura Sireno, Virgilio Pérez, Consuelo Borrás

**Affiliations:** 1 MiniAging/Freshage Research Group, Department of Physiology, Faculty of Medicine, University of Valencia, Centro de Investigación Biomédica en Red Fragilidad y Envejecimiento Saludable-Instituto de Salud Carlos III (CIBERFES-ISCIII), Institute of Health Research-INCLIVA, 46010 Valencia, Spain; cristina.mas@uv.es (C.M.-B.); javier.huete@uv.es (J.H.-A.); marta.arnal@uv.es (M.A.-F.); 2 Unit of Biology and Genetics of Movement, Department of Movement, Human and Health Sciences, University of Rome Foro Italico, 00135 Rome, Italy; l.sireno@studenti.uniroma4.it; 3 Department of Applied Economics (Quantitative Methods), Faculty of Economics, University of Valencia, 46022 Valencia, Spain; virgilio.perez@uv.es

**Keywords:** extracellular vesicles, miRNAs, oxidative stress, ROS, epigenetics

## Abstract

Extracellular vesicles (EVs) are emerging as key regulators of cellular communication, with increasing evidence supporting their role in oxidative stress (OS) modulation. In particular, the miRNA cargo of EVs plays a crucial role in mitigating OS and promoting redox balance through both direct antioxidant effects and epigenetic regulation. This study aimed to evaluate the impact of EVs on OS markers, influenced by their miRNA-mediated effects and potential epigenetic modifications in target cells. A systematic literature search was conducted following the Preferred Reporting Items for Systematic Reviews and Meta-Analysis (PRISMA) guidelines to identify studies reporting the effects of EVs on OS parameters. A meta-analysis was performed on key OS biomarkers, including reactive oxygen species (ROS), superoxide dismutase (SOD), glutathione (GSH), and malondialdehyde (MDA). The heterogeneity of EV isolation and characterization techniques was also analyzed. The included studies demonstrated that EVs exert significant antioxidant effects by reducing ROS levels, increasing SOD activity and GSH levels, and lowering MDA levels. These effects were largely attributed to EV-miRNAs, which induce epigenetic modifications that modulate redox-related signaling pathways. However, the variability in EV isolation methods and characterization approaches highlights the need for standardization to improve data comparability. Despite their therapeutic potential, this significant heterogeneity in EV research remains a barrier to translation. Moreover, further exploration of epigenetic mechanisms is essential to fully harness their benefits for OS-related diseases.

## 1. Introduction

Epigenetics is an evolving field of study that explores the molecular mechanisms influencing gene expression and heritable traits without altering the underlying DNA sequence. The term “epigenetics” refers to changes in gene activity that do not involve alterations to the DNA sequence itself. Instead, it involves modifications to the DNA structure or the proteins with which DNA interacts. Epigenetics primarily regulates gene expression, determining which genes are turned on or off in a particular cell at a given time. This regulation is crucial for the development, differentiation, and functioning of various cell types within an organism. Three main epigenetic mechanisms exist: DNA methylation, histone modification, and non-coding RNA molecules [1].

DNA methylation involves adding methyl groups to the cytosine bases of DNA. This modification usually occurs at specific CpG island sequences (CGI; genomic regions that contain a high frequency of cytosine and guanine nucleotides connected by a phosphodiester bond) and is associated with gene silencing. When a gene is methylated, it is less likely to be transcribed and, therefore, less likely to be expressed [2]. Histones are proteins that package DNA into a compact structure called chromatin. Chemical modifications to histones, such as acetylation, methylation, phosphorylation, and ubiquitination, can alter the chromatin structure and influence gene expression. For example, acetylation of histones is generally associated with gene activation, while methylation can either activate or repress gene expression, depending on the specific context [3]. Non-coding RNAs, such as microRNAs (miRNAs) and long non-coding RNAs (lncRNAs), play a role in post-transcriptional regulation. They can bind to messenger RNAs (mRNAs) and either promote their degradation or inhibit their translation into proteins. This, in turn, affects the amount of protein produced from a particular gene [4].

Extracellular vesicles (EVs) have emerged as key mediators of intercellular communication, transferring bioactive molecules such as proteins, lipids, and nucleic acids, including miRNAs and lncRNAs [5]. In recent years, growing evidence has highlighted the involvement of EV-derived miRNAs in redox biology, suggesting that these molecules can influence oxidative stress (OS) responses and epigenetic modifications in target cells [5].

Changes in the redox state play a crucial role in regulating various cellular processes, including cell growth, differentiation, apoptosis, and immune response. OS, resulting from an imbalance between reactive oxygen species (ROS) production and antioxidant defenses, is a key contributor to aging and various pathological conditions, including neurodegenerative diseases, cancer, and cardiovascular disorders [6]. Cells have evolved sophisticated mechanisms to counteract oxidative damage, including enzymatic (e.g., superoxide dismutase, catalase, glutathione peroxidase) and non-enzymatic antioxidant systems. Emerging research indicates that EVs can modulate these redox processes by delivering miRNAs that regulate key components of OS responses. Specifically, miRNAs carried by EVs have been shown to target mRNAs encoding redox-related enzymes, thereby influencing ROS levels, lipid peroxidation (e.g., malondialdehyde [MDA] production), and overall redox homeostasis [7].

The interplay between EV-mediated miRNA transfer and redox regulation also extends to epigenetic modifications, which influence gene expression without altering the DNA sequence. miRNAs are well recognized as epigenetic regulators, capable of modulating DNA methylation, histone modifications, and chromatin remodeling [8]. By limiting the expression of antioxidant genes and redox-sensitive transcription factors, EV-derived miRNAs may contribute to cellular adaptation to OS [9], with potential implications for disease progression and therapeutic strategies.

This systematic review aims to comprehensively analyze the current evidence supporting the role of EV-carried miRNAs in redox homeostasis. Specifically, we will explore their capacity to regulate post-transcriptional processes, enhance antioxidant defenses, and mitigate oxidative damage in recipient cells. Understanding these mechanisms may open new avenues for targeting OS-related diseases through EV-based strategies.

## 2. Materials and Methods

This systematic review was prepared in accordance with the Preferred Reporting Items for Systematic Reviews and Meta-Analysis (PRISMA) guidelines for systematic reviews [10]. The protocol of this study was developed a priori, peer-reviewed, registered, and published in the Open Science Framework (OSF; protocol ID: s2bc4).

### 2.1. Literature Search Strategy

A literature search was performed in PubMed and conducted according to standard systematic review methodology to ensure transparency and reproducibility. The following keywords and Boolean operators were used: (“extracellular vesicles” OR “exosomes” OR “microvesicles”) AND (“ROS” OR “redox” OR “oxidative stress”) AND (“epigenetics” OR “DNA methylation” OR “histone” OR “non-coding RNA”).

### 2.2. Study Selection: Inclusion and Exclusion Criteria

The inclusion criteria consisted of peer-reviewed original research studies published in the last 10 years (2015–2025), written in English, and available as free full-text publications. Keywords were required to appear in each article’s title and/or abstract. The exclusion criteria were as follows: non-primary or secondary research (including literature reviews, meta-analyses, letters to the editor, commentaries, preprints, poster/conference presentations or abstracts, dissertations, protocols, and ongoing project reports) published before 2015, not written in the English language, or lacking available full text.

**Figure 1:**
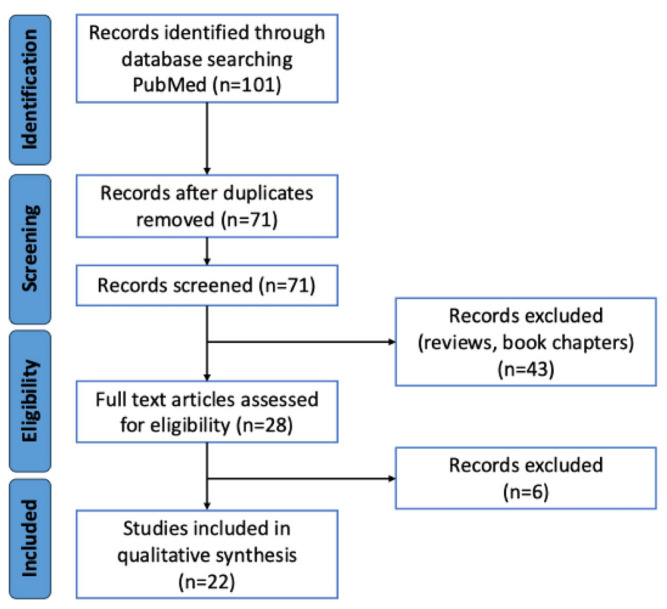
PRISMA flow diagram of the selected studies.

Figure 1 illustrates the process of identifying and selecting eligible studies. Initially, 101 records were retrieved. Among these, 30 duplicates were removed. As a result, 71 unique records remained that were assessed for eligibility after screening. Of these, 43 were excluded because they were not original studies, and 6 more were excluded because they did not report oxidative stress-related or epigenetic-related data relevant to this study. Ultimately, 22 studies were included in the analysis [11,12,13,14,15,16,17,18,19,20,21,22,23,24,25,26,27,28,29,30,31,32].

### 2.3. Risk of Bias Assessment

The quality of the included studies was assessed using the Risk of Bias in Non-Randomized Studies of Interventions (ROBINS-I) tool for both in vivo and in vitro studies [33]. Studies were categorized as low, moderate, or severe risk of bias based on selection bias, confounding variables, blinding, and outcome reporting.

The assessment of the risk of bias revealed that six studies were identified as low risk, making them the most reliable sources for the meta-analysis; fourteen studies had moderate risk, indicating that their results should be interpreted with some caution; and two studies were classified as serious risk, raising concerns about their reliability. Sensitivity analysis should consider excluding these studies (see Table 1). Most studies exhibited confounding and selective reporting issues, emphasizing the need for standardized methodologies in EV research. Low-risk studies (e.g., Fafián-Labora et al. (2020) [14], Sanz-Ros et al. (2022) [21], Della Rocca et al. (2024) [28], and Gyorgy et al. (2025) [31]) were prioritized for conclusion. Serious-risk studies (Wang et al. (2019) [12] and Hussain et al. (2023) [24]) were carefully evaluated. Overall, while most studies support the beneficial effects of EVs, methodological variability remains a challenge, reinforcing the need for standardized protocols in future research.

### 2.4. Analysis and Data Synthesis

The final selection of studies was reviewed in detail. The extracted information included the author, year of publication, experimental model, EV source, EV type, isolation technique, characterization methods, EV cargo content, and downstream effects on target cells, focusing on OS markers and epigenetic modifications.

A meta-analysis was conducted using a meta-R package (version 8.0-2) to assess differences in EV size and concentration between experimental and control groups [34]. The metacont function was used to analyze continuous data (ROS levels, SOD levels, SOD activity, GSH levels, and MDA levels), specifying sample sizes, means, and standard deviations for both experimental and control groups.

The effect size was estimated using the Standardized Mean Difference (SMD), with Hedges’ g as the selected method. This approach is preferred in studies with small sample sizes, as it corrects the bias in SMD estimation. Given the expected heterogeneity among studies, a random-effects model was applied, which assumes that differences between studies may arise from both within-study and between-study variability, thereby providing a more generalizable effect estimate [35].

Additionally, a prediction interval was calculated, providing an estimate of the range in which effects in future similar studies are expected to fall. This offers insights into the generalizability of the findings beyond the analyzed studies. A fixed-effects model was not computed, as it assumes homogeneity across studies, which was deemed inappropriate given the expected variability in methodologies and EV characteristics.

## 3. Results

### 3.1. Study Results Synthesis

**Figure 2:**
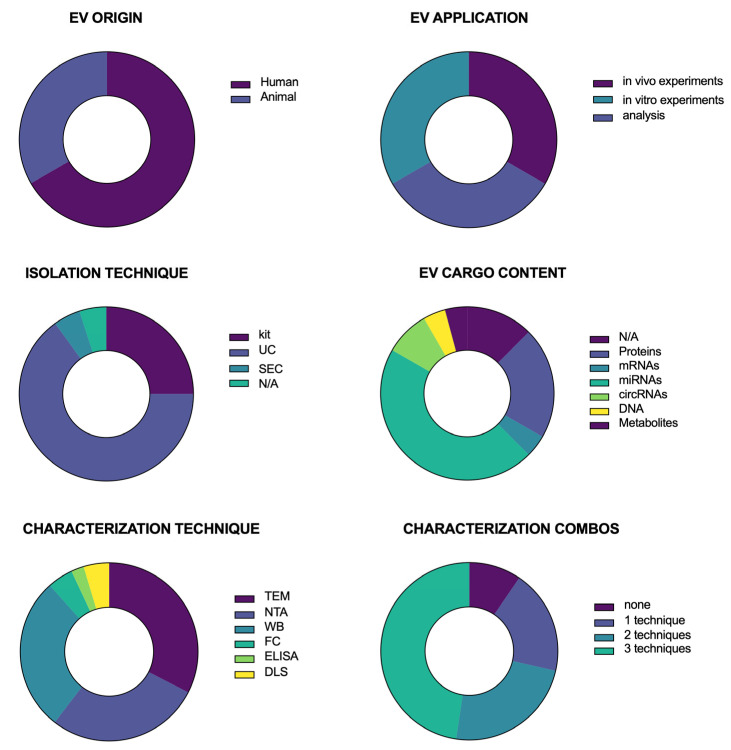
Summary of the reported data by the selected studies.

Figure 2 presents a series of donut charts summarizing the distribution of key characteristics across the studies included in this systematic review. Each panel represents a different categorical variable, with color-coded segments corresponding to the proportion of studies within each category. EVs were predominantly of animal (non-primate) origin rather than human origin. EVs were mostly isolated by ultracentrifugation (UC) or using commercial kits, although some papers used size-exclusion chromatography (SEC) or did not report any information about the isolation technique.

EVs were used for in vitro and in vivo experiments or cargo content analysis. Regarding EV cargo content, some studies did not analyze it. In contrast, most assessed their nucleic acid content (mRNA, miRNA, circRNA, and DNA), followed by the analysis of protein and metabolic cargo. Following isolation, EVs were characterized. The most commonly used characterization techniques were transmission electron microscopy (TEM), nanoparticle tracking analysis (NTA), and Western Blotting (WB). Some studies also performed Flow Cytometry (FC), Enzyme-linked Immunosorbent Analysis (ELISA), or Dynamic Light Scattering (DLS). Importantly, none of these techniques is sufficient on its own. Thus, many papers offered a combination of two or even three techniques.

These data illustrate the diversity of methodological approaches and thematic foci within the reviewed literature, providing insights into potential gaps and areas of focus in the field.

### 3.2. EV Size and Concentration: NTA Data Summary

**Figure 3:**
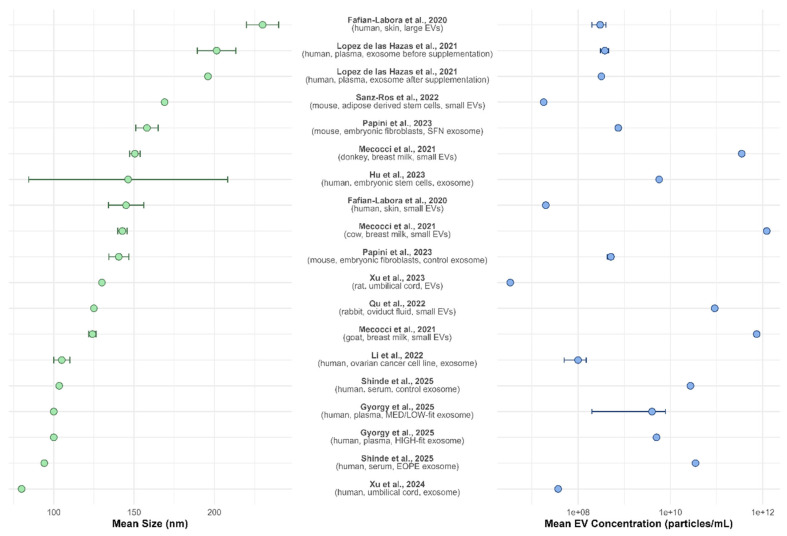
Summary of the NTA analysis of EVs across different studies. The left panel shows the distribution of the mean EV size (in nanometers), while the right panel represents the mean EV concentration (in particles/mL). Each point indicates the reported mean value for a given study, with error bars representing the range from mean ± standard deviation.

Figure 3 presents a double plot summarizing the nanoparticle tracking analysis (NTA) data reported across multiple studies, including the mean EV size (nm) and concentration (particles/mL). The left panel illustrates the mean EV size with 95% confidence intervals, revealing variability across different sources and isolation methods. The reported EV sizes range from approximately 90 to over 250 nm, with most studies clustering around 100–150 nm. Larger EVs were observed in studies focusing on skin-derived vesicles [14], plasma [17], and adipose-derived stem cell EVs [21]. In contrast, smaller vesicles were reported for umbilical cord-derived exosomes [30].

The right panel of the figure displays EV concentration, which spans several orders of magnitude, from approximately 10^7^ to 10^12^ particles/mL. Notably, umbilical cord-derived exosomes [30], skin-derived vesicles [14], and adipose-derived stem cell EVs [21] exhibited the lowest concentrations. In contrast, higher EV concentrations were found in studies analyzing breast milk from different species [18]. EV concentration variation may reflect differences in sample type, isolation techniques, and experimental conditions across studies.

Together, these data highlight the heterogeneity in EV size and concentration among different biological sources and underscore the need for standardized isolation techniques and characterization methodologies in EV research.

### 3.3. Effect of EV Treatment on ROS Levels

**Figure 4:**
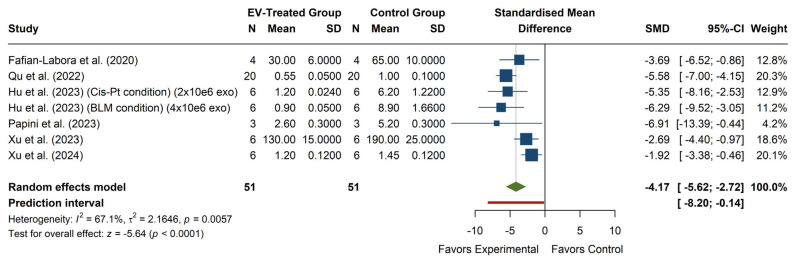
Forest plot of the effect of EVs on ROS levels. Color legend: Standardized Mean Difference of each article (blue), Average Standardized Mean Difference of all articles (green), Prediction Interval (red).

The analysis of ROS levels (see Figure 4) shows a significant overall effect, with an SMD of −4.17 (95% CI: −5.62, −2.72), *p* < 0.0001, indicating that EV treatment significantly reduces ROS levels compared to the control group. Individual studies show consistent effects in favor of EV treatment, with all 95% confidence intervals falling within the negative range, reinforcing the robustness of the observed effect. The heterogeneity analysis revealed moderate-to-high variability among studies (I^2^ = 67.1%, *p* = 0.0057), suggesting that methodological or biological factors may contribute to the observed differences in effect sizes. Additionally, the prediction interval (−8.20, −0.14) indicates that, although most future studies are likely to report a reduction in ROS with EV treatment, the effect may be smaller or even non-significant in some cases.

### 3.4. Effect of EV Treatment on SOD Levels

**Figure 5:**
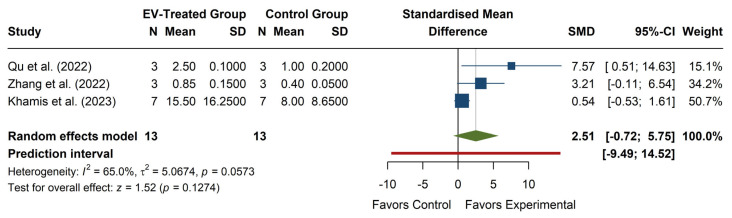
Forest plot of the effect of EVs on SOD levels. Color legend: Standardized Mean Difference of each article (blue), Average Standardized Mean Difference of all articles (green), Prediction Interval (red).

The meta-analysis of SOD levels (see Figure 5) revealed a non-significant overall effect, with an SMD of 2.51 (95% CI: −0.72, 5.75), z = 1.52, *p* = 0.1274, suggesting that EV treatment does not significantly increase SOD levels compared to the control. The confidence interval includes zero, indicating that the effect size is uncertain and could range from negative to strongly positive. Individual studies show high variability in effect sizes, with Qu et al. (2022) [20] reporting a strong positive effect (SMD = 7.57, 95% CI: 0.51, 14.63), while Zhang et al. (2022) [22] and Khamis et al. (2023) [25] report more minor, non-significant effects. The heterogeneity was moderate to high (I^2^ = 65.0%, *p* = 0.0573), with τ^2^ = 5.07, indicating considerable variation between studies. The prediction interval (−9.49, 14.52) suggests that future studies could observe a wide range of possible effects, from a substantial decrease to a strong increase in SOD levels. Overall, while some evidence suggests a potential increase in SOD with EV treatment, the high heterogeneity and lack of statistical significance limit the interpretability of the results.

### 3.5. Effect of EV Treatment on SOD Activity

**Figure 6:**
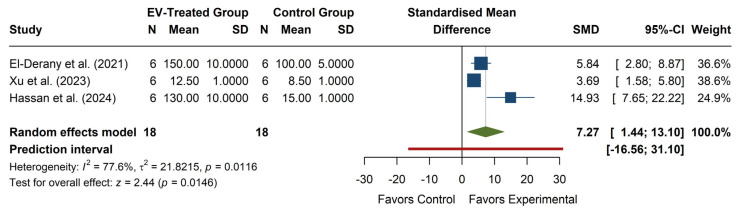
Forest plot of the effect of EVs on SOD activity. Color legend: Standardized Mean Difference of each article (blue), Average Standardized Mean Difference of all articles (green), Prediction Interval (red).

The meta-analysis of SOD activity (see Figure 6) revealed a significant overall effect, with an SMD of 7.27 (95% CI: 1.44, 13.10), z = 2.44, *p* = 0.0146, indicating that EV treatment significantly increases SOD activity compared to the control. Individual studies show positive effects, with Hassan et al. (2024) [29] reporting the largest SMD (14.93, 95% CI: 7.65, 22.22), while El-Derany et al. (2021) [15] and Xu et al. (2023) [27] reported more moderate increases. The heterogeneity was high (I^2^ = 77.6%, *p* = 0.0116), with τ^2^ = 21.82, suggesting substantial variability in effect sizes across studies. The prediction interval (−16.56, 31.10) indicates a wide range of potential future outcomes, including possible adverse effects, although the overall trend favors increased SOD activity with EV treatment.

### 3.6. Effect of EV Treatment on GSH Levels

**Figure 7:**
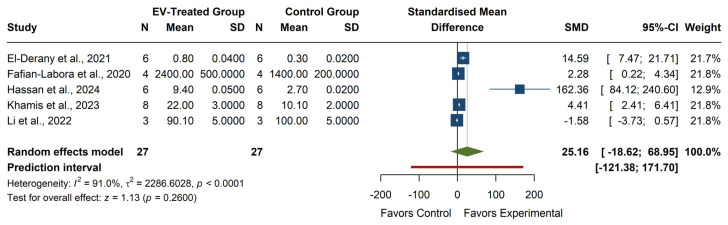
Forest plot of the effect of EVs on GSH levels. Color legend: Standardized Mean Difference of each article (blue), Average Standardized Mean Difference of all articles (green), Prediction Interval (red).

The meta-analysis of GSH levels (see Figure 7) showed a non-significant overall effect, with an SMD of 25.16 (95% CI: −18.62, 68.95), z = 1.13, *p* = 0.2600, indicating that EV treatment does not lead to a consistent increase in GSH levels. Individual studies exhibited high variability, with Hassan et al. (2024) [29] reporting a considerable effect (SMD = 162.36, 95% CI: 84.12, 240.60), while Li et al. (2022) [19] showed a negative, non-significant effect (SMD = −1.58, 95% CI: −3.73, 0.57). The heterogeneity was high (I^2^ = 91.0%, *p* < 0.0001, τ^2^ = 2286.60), reflecting substantial variation across studies. The prediction interval (−121.38, 171.70) suggests a broad range of potential outcomes in future studies, from significant negative to extreme positive effects. The lack of statistical significance and the high heterogeneity indicate that the impact of EVs on GSH levels remains inconclusive.

### 3.7. Effect of EV Treatment on MDA Levels

**Figure 8:**
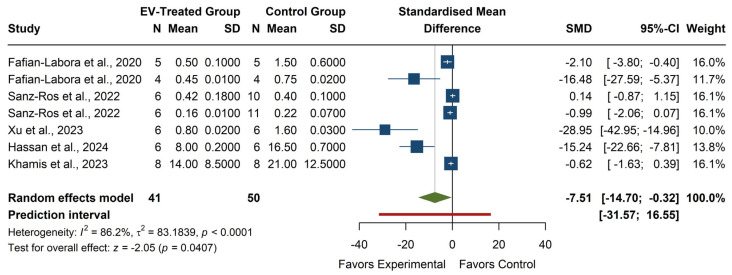
Forest plot of the effect of EVs on MDA levels. Color legend: Standardized Mean Difference of each article (blue), Average Standardized Mean Difference of all articles (green), Prediction Interval (red).

The meta-analysis of MDA levels (see Figure 8) revealed a significant overall effect, with an SMD of −7.51 (95% CI: −14.70, −0.32), z = −2.05, *p* = 0.0407, suggesting that EV treatment significantly reduces MDA levels compared to the control group. Individual studies showed high variability in effect sizes, with Xu et al. (2023) [27] reporting the most significant reduction (SMD = −28.95, 95% CI: −42.95, −14.96), while some studies, such as Sanz-Ros et al. (2022) [21] and Khamis et al. (2023) [25], reported small or non-significant effects. The heterogeneity was high (I^2^ = 86.2%, *p* < 0.0001, τ^2^ = 83.18), indicating substantial differences across studies. The prediction interval (−31.57, 16.55) suggests that future studies might observe a wide range of effects, including potential increases in MDA. Despite the significant overall effect, the considerable heterogeneity highlights the need for further investigation into factors influencing the impact of EV treatment on MDA levels.

## 4. Discussion

The findings of this systematic review with meta-analysis highlight considerable variability in EV isolation methods and characterization across different studies. The selected studies used several isolation techniques, such as ultracentrifugation (with varying forces of g and times), size exclusion chromatography, and commercial kits. Since there is no specific marker for EVs, most studies used a combination of two or more methods to characterize their EVs, such as nanoparticle tracking analysis (NTA), Western Blot (WB), Flow Cytometry (FC), Mass Spectrometry (MS), and transmission electron microscopy (TEM).

The NTA data summary illustrates that EV size and concentration can vary significantly depending on the biological source, isolation technique, and analytical method used. This heterogeneity underscores the urgent need for standardized protocols to ensure reproducibility and comparability across studies. The lack of uniformity in EV isolation and characterization remains a significant challenge in the field, limiting the translational potential of EV-based interventions. Efforts to establish consensus guidelines, such as those proposed by the International Society for Extracellular Vesicles (ISEV) [36], should be further refined and widely adopted to enhance the robustness of EV research.

Despite methodological discrepancies, our meta-analysis confirms that EV cargo transfer, particularly miRNAs, exerts beneficial effects on target cells. Specifically, EVs contribute to redox homeostasis by reducing ROS levels, enhancing SOD activity, increasing GSH levels, and lowering MDA levels. These findings suggest that EVs possess potent antioxidant properties, which could be leveraged for therapeutic applications in conditions associated with oxidative stress, such as aging, neurodegenerative disorders, and metabolic diseases [37]. The ability of EVs to modulate OS pathways aligns with previous evidence that demonstrates their role in cellular protection, tissue repair, and metabolic regulation.

One of the key mechanisms by which EVs exert their antioxidant effects is through epigenetic modifications induced by their miRNA cargo. Recipient cells can internalize EV-derived miRNAs, which regulate gene expression post-transcriptionally by targeting messenger RNAs (mRNAs) involved in OS responses. Indeed, several miRNAs carried by EVs have been implicated in the regulation of antioxidant defense systems. For example, specific miRNAs can modulate the expression of nuclear factor erythroid 2-related factor 2 (NRF2), a master regulator of the antioxidant response, by either directly targeting its inhibitors (e.g., KEAP1) or activating NRF2-responsive genes involved in detoxification and ROS scavenging [38,39,40].

However, evidence suggests that these miRNAs can induce more stable, long-term cellular adaptations through epigenetic modifications, including DNA methylation, histone modifications, and chromatin remodeling. EV-miRNAs have been shown to regulate histone deacetylases and DNA methyltransferases, which influence chromatin accessibility and the transcriptional activation of genes coding for antioxidant enzymes, such as superoxide dismutase (SOD), catalase (CAT), and glutathione peroxidase (GPx) [37,41,42,43].

Furthermore, EV-mediated epigenetic modifications extend beyond acute responses to OS, promoting cellular reprogramming toward a more resilient, antioxidant-enhanced phenotype. For instance, some EV-miRNAs downregulate pro-oxidant and pro-inflammatory pathways by silencing key genes in NF-κB and TNF-α signaling, leading to sustained reductions in OS and inflammatory damage [44,45,46]. In stem and progenitor cells, EV-mediated epigenetic modifications have also been linked to improved mitochondrial function and biogenesis, further reinforcing cellular defense mechanisms against OS [47].

These findings suggest that EVs do not merely deliver transient antioxidant benefits but can induce long-lasting changes in recipient cells through epigenetic regulation. This concept is particularly relevant for aging and age-related diseases, where the accumulation of oxidative damage contributes to cellular dysfunction. By modulating epigenetic landscapes, EVs may represent a promising tool for restoring redox balance and enhancing tissue homeostasis over time.

## 5. Limitations

While our findings provide strong evidence for the beneficial effects of EVs on redox balance, several limitations should be acknowledged.

First, the heterogeneity in EV isolation and characterization methods introduces variability in the reported outcomes, which may affect the overall strength of the conclusions. Differences in isolation techniques, including ultracentrifugation, size-exclusion chromatography, and kit-based methods, can influence EV purity, yield, and cargo composition, potentially confounding the reported effects.

Second, the included studies vary regarding EV source, donor characteristics, and experimental conditions (in vivo/in vitro). Factors such as age, health status, and environmental influences may affect EV composition and bioactivity, limiting the generalizability of the findings. Additionally, most studies included in this review were conducted in preclinical models or in vitro settings, which may not fully recapitulate the complexity of clinical conditions. Further validation in clinical studies is necessary to establish the translational relevance of these findings.

Third, although our meta-analysis identified significant antioxidant effects of EVs, potential publication bias and selective reporting cannot be ruled out. Studies reporting null or negative findings may be underrepresented in the literature, which could lead to the overestimation of EV efficacy. Future systematic reviews should incorporate bias assessment tools and aim to include unpublished or negative results more comprehensively.

Finally, the variability in the methodologies used for functional assays, including differences in OS markers and measurement techniques, adds another layer of complexity. Standardized approaches for assessing EV function in OS models are needed to improve cross-study comparisons and data reproducibility.

## 6. Conclusions

In conclusion, this systematic review with meta-analysis supports the role of EVs as key modulators of OS through their miRNA cargo. Beyond their immediate effects on ROS scavenging and antioxidant enzyme activity, EVs induce long-term cellular adaptations through epigenetic regulation. This ability to modulate the expression of redox-related genes at the epigenetic level suggests that EVs may serve as powerful therapeutic tools for OS-related disorders. However, the standardization of EV isolation and characterization techniques, as well as more in-depth mechanistic studies on EV-induced epigenetic modifications, are imperative to fully harness their potential. Future research should validate these findings in clinical settings and explore strategies to optimize EV-based interventions for enhancing cellular resilience against oxidative damage.

## Figures and Tables

**Table 1:** Risk of bias assessment of the selected studies.

Study	Confounding	Selection Bias	Classification of Interventions	Deviations from Intended Interventions	Missing Data	Measurement of Outcomes	Selective Reporting	Overall Risk
Pavanello et al. (2016) [11]	Moderate	Low	Low	Moderate	Low	Moderate	Moderate	Moderate
Wang et al. (2019) [12]	Serious	Moderate	Low	Serious	Moderate	Moderate	Serious	Serious
Xu et al. (2019) [13]	Moderate	Moderate	Moderate	Low	Low	Low	Moderate	Moderate
Fafián-Labora et al. (2020) [14]	Low	Low	Low	Low	Low	Low	Low	Low
El-Derany et al. (2021) [15]	Low	Low	Low	Low	Moderate	Moderate	Moderate	Moderate
Homme et al. (2021) [16]	Moderate	Moderate	Low	Moderate	Low	Low	Moderate	Moderate
Lopez de las Hazas et al. (2021) [17]	Moderate	Low	Low	Moderate	Low	Moderate	Moderate	Moderate
Mecocci et al. (2021) [18]	Low	Low	Low	Low	Low	Low	Moderate	Low
Li et al. (2022) [19]	Moderate	Moderate	Low	Moderate	Moderate	Moderate	Moderate	Moderate
Qu et al. (2022) [20]	Moderate	Low	Low	Moderate	Low	Moderate	Moderate	Moderate
Sanz-Ros et al. (2022) [21]	Low	Low	Low	Low	Low	Low	Low	Low
Zhang et al. (2022) [22]	Moderate	Low	Low	Moderate	Low	Moderate	Moderate	Moderate
Hu et al. (2023) [23]	Moderate	Low	Low	Moderate	Low	Moderate	Moderate	Moderate
Hussain et al. (2023) [24]	Serious	Moderate	Moderate	Serious	Moderate	Moderate	Serious	Serious
Khamis et al. (2023) [25]	Low	Low	Low	Low	Low	Low	Low	Low
Papini et al. (2023) [26]	Moderate	Low	Low	Moderate	Moderate	Low	Moderate	Moderate
Xu et al. (2023) [27]	Low	Low	Moderate	Low	Low	Moderate	Moderate	Moderate
Della Rocca et al. (2024) [28]	Low	Low	Low	Moderate	Low	Low	Low	Low
Hassan et al. (2024) [29]	Moderate	Low	Low	Moderate	Moderate	Moderate	Moderate	Moderate
Xu et al. (2024) [30]	Moderate	Moderate	Low	Low	Low	Moderate	Moderate	Moderate
Gyorgy et al. (2025) [31]	Low	Low	Low	Low	Low	Low	Low	Low
Shinde et al. (2025) [32]	Moderate	Moderate	Low	Moderate	Low	Moderate	Moderate	Moderate

## Data Availability

Not applicable.
